# Supplementary material for: Artificial Sporulation Induction (ASI) by kinA Overexpression Affects the Proteomes and Properties of Bacillus subtilis Spores
Source: Int J Mol Sci. 2020 Jun 17;21(12):4315. doi: 10.3390/ijms21124315 (PMC7352945; doi:10.3390/ijms21124315)
Supplement: Supplementary file 1 [file ijms-21-04315-s001.zip › supplementary data.pdf]

Supplementary Data

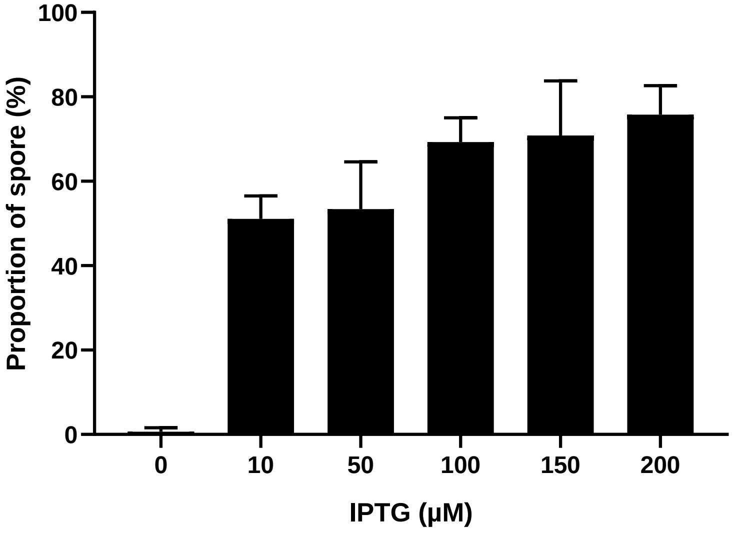

Figure S1. The sporulation efficiency of the mutant strain with induction of *kinA* at different concentrations of IPTG after 8 h of glucose dilution.

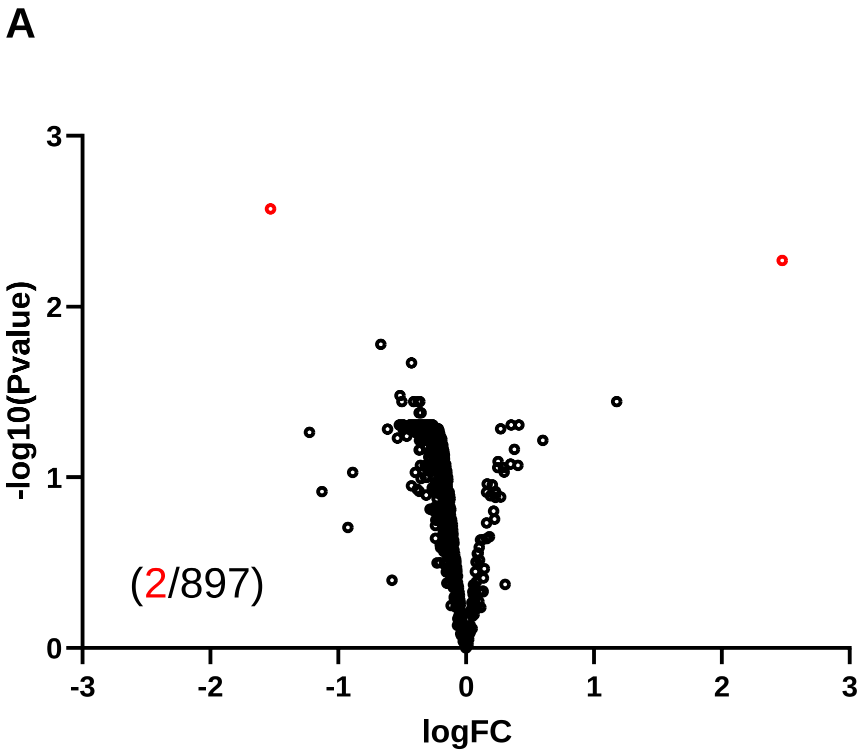

B

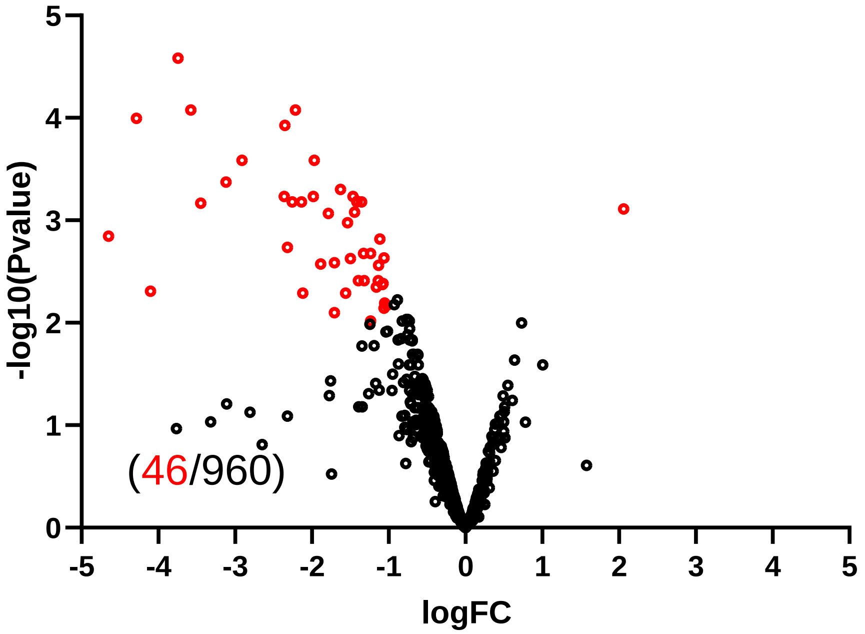

C

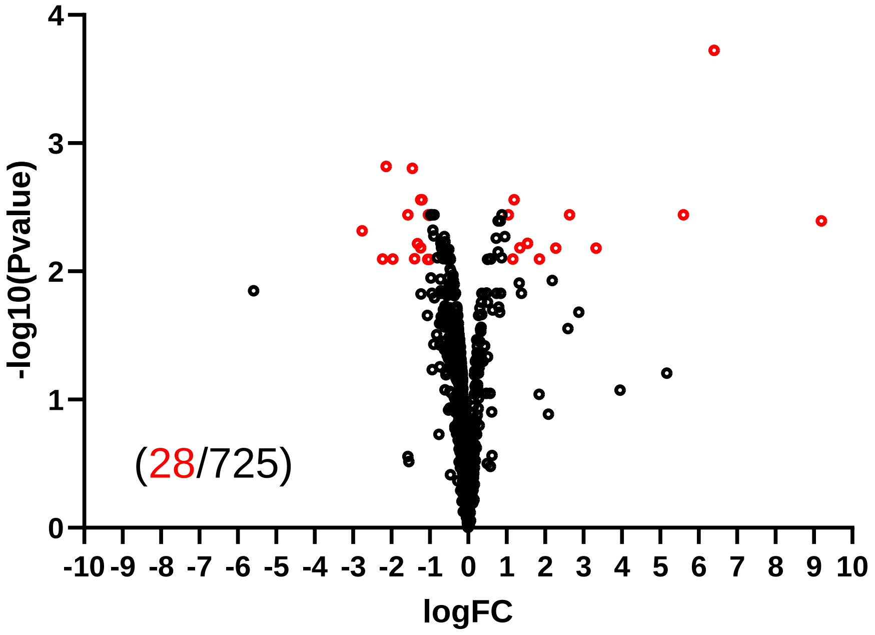

Figure S2. Volcano plots of proteomic comparison of spores or cells. X-axis indicates the average of log2 isotopic ratios from the replicates. Negative values indicate downregulation and positive

values indicate upregulation, Y axis is –$\log_{10}$ p-value. Dots in red indicate differentially expressed proteins. Dots in black indicate proteins that are not significant changed in protein expression. Numbers in red and black in brackets are the number of differentially expressed proteins and total quantified proteins, respectively. **(A)** WT+ spores are compared to WT- sores. **(B)** M- cells are compared to WT- cells. **(C)** M- spores are compared to WT- spores.

A

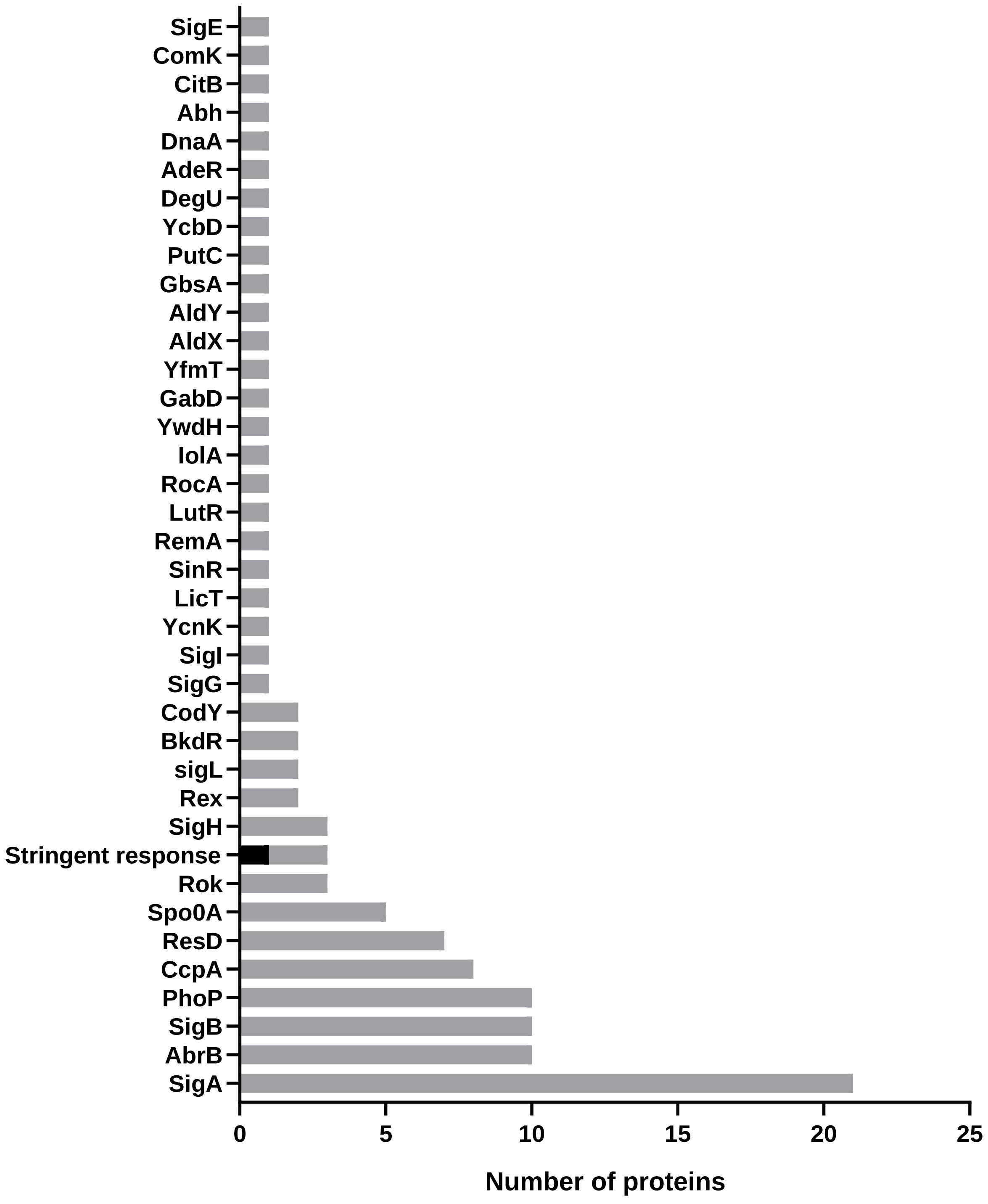
SigE
ComK
CitB
Abh
DnaA
AdeR
DegU
YcbD
PutC
GbsA
AldY
AldX
YfmT
GabD
YwdH
IolA
RocA
LutR
RemA
SinR
LicT
YcnK
SigI
SigG
CodY
BkdR
sigL
Rex
SigH
Stringent response
Rok
Spo0A
ResD
CcpA
PhoP
SigB
AbrB
SigA
0
5
10
15
20
25
Number of proteins

B

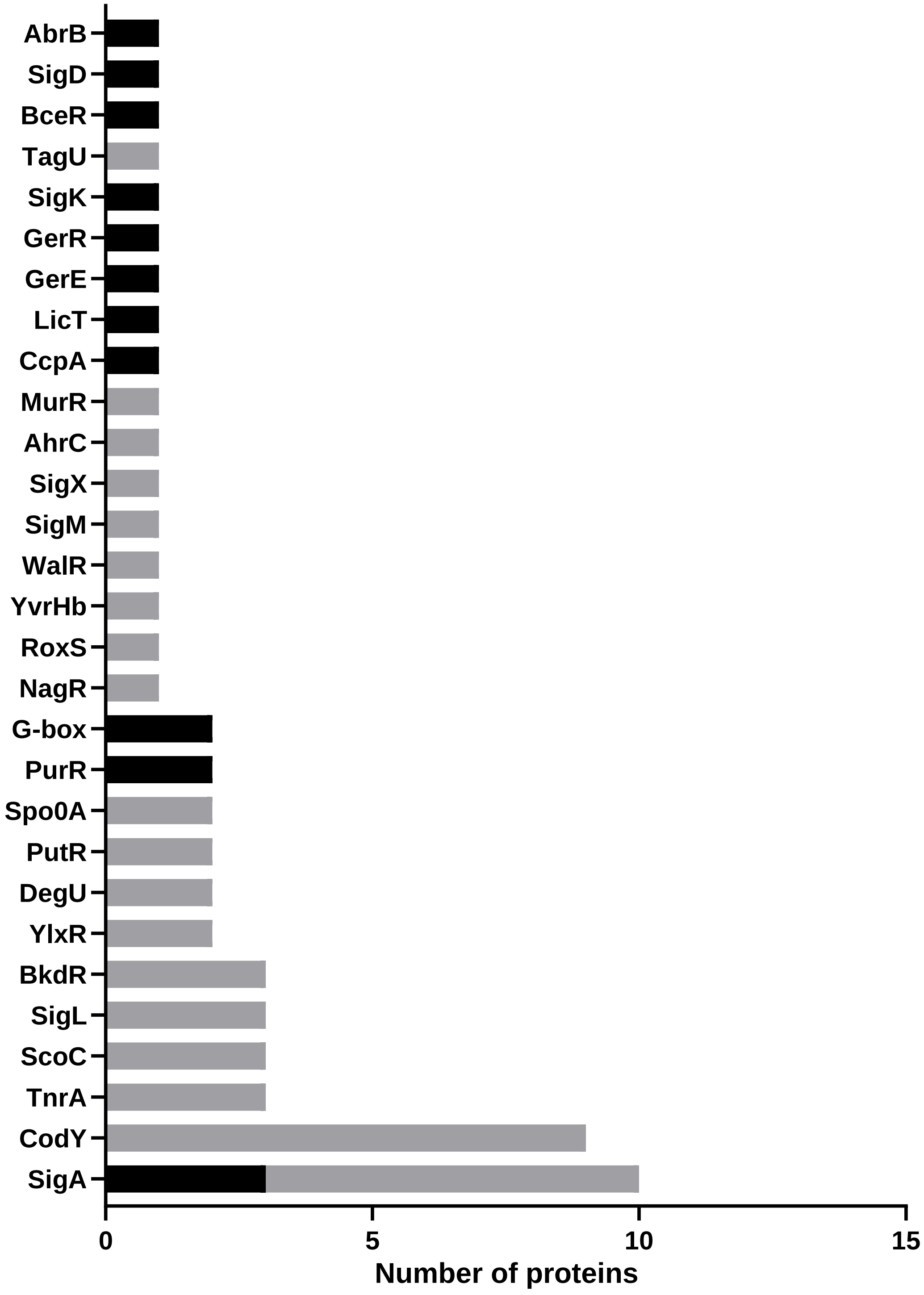

AbrB
SigD
BceR
TagU
SigK
GerR
GerE
LicT
CcpA
MurR
AhrC
SigX
SigM
WalR
YvrHb
RoxS
NagR
G-box
PurR
Spo0A
PutR
DegU
YlxR
BkdR
SigL
ScoC
TnrA
CodY
SigA
0
5
10
15
Number of proteins

C

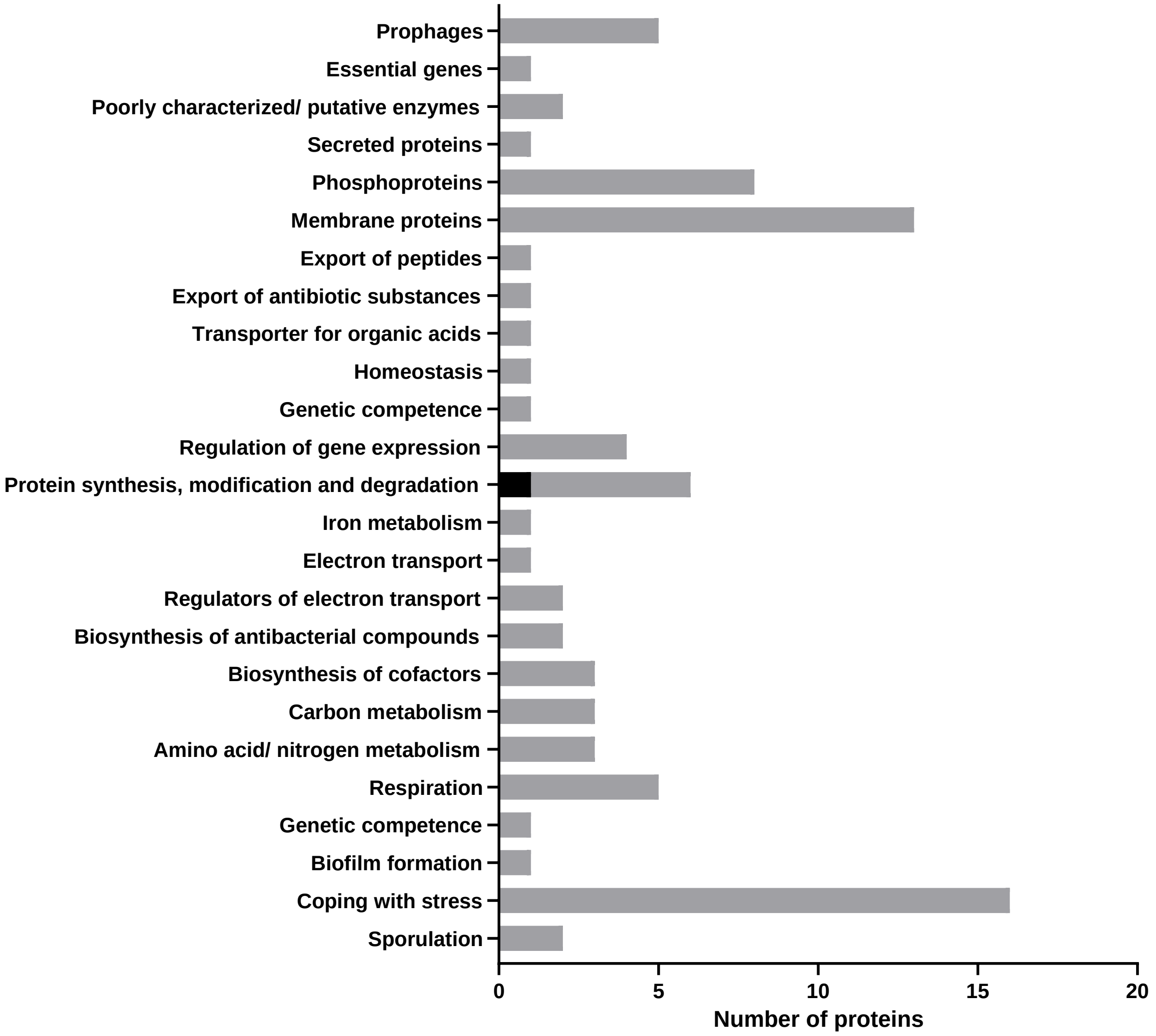

Prophages
Essential genes
Poorly characterized/ putative enzymes
Secreted proteins
Phosphoproteins
Membrane proteins
Export of peptides
Export of antibiotic substances
Transporter for organic acids
Homeostasis
Genetic competence
Regulation of gene expression
Protein synthesis, modification and degradation
Iron metabolism
Electron transport
Regulators of electron transport
Biosynthesis of antibacterial compounds
Biosynthesis of cofactors
Carbon metabolism
Amino acid/ nitrogen metabolism
Respiration
Genetic competence
Biofilm formation
Coping with stress
Sporulation
0
5
10
15
20
Number of proteins

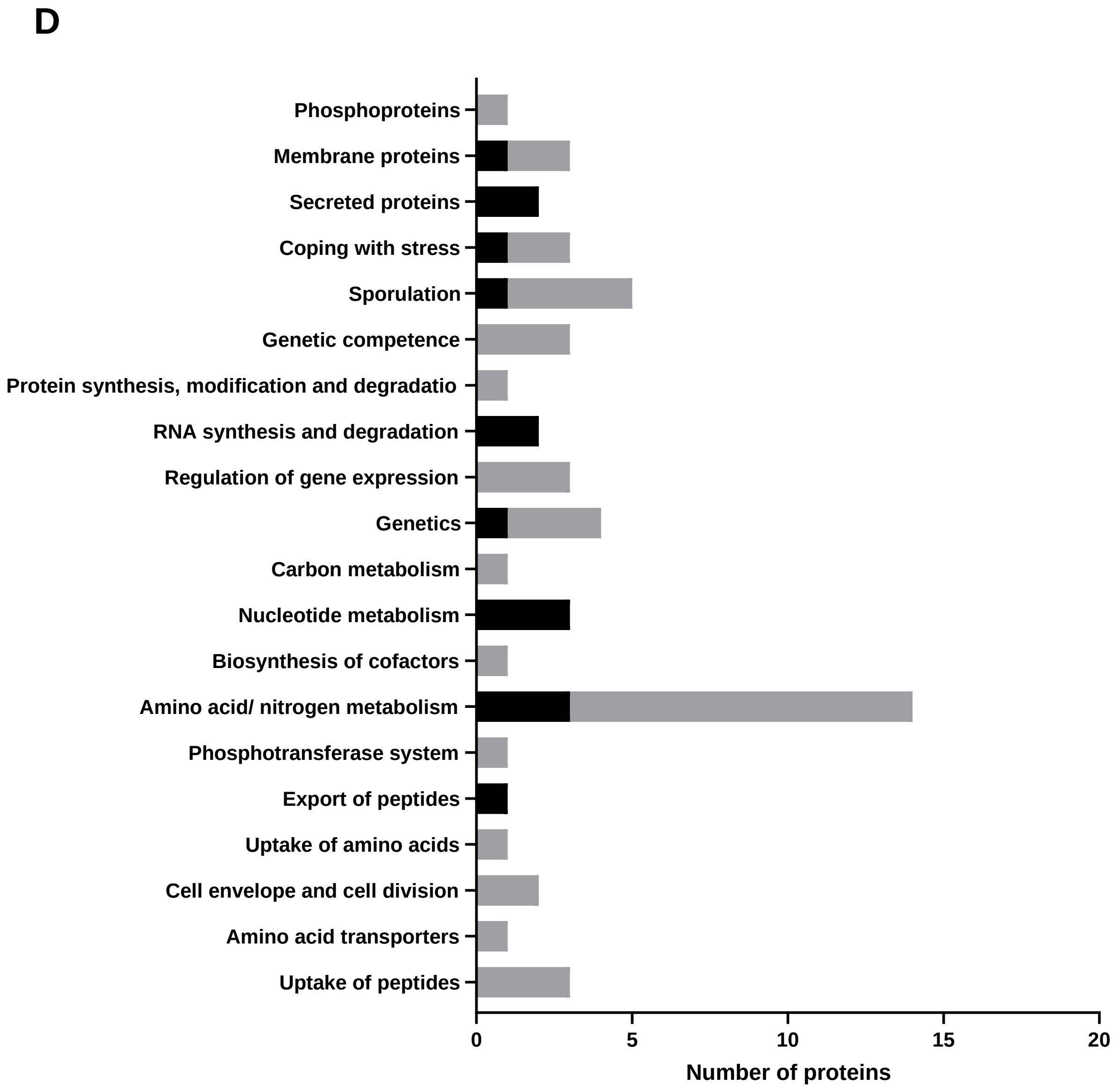

Figure S3. Regulators and functional classification of differentially expressed proteins. X-axis indicates the number of proteins classified into a regulatory or functional categories on the y-axis. Note that every protein could occupy more than one regulatory or functional category. Bars in black and gray indicate number of upregulated and downregulated proteins, separately (A) Regulators of differentially expressed proteins in M- cells compared to WT- cells. (B) Regulators of differentially expressed proteins in M- spores compared to WT- spores. (C) and (D) are the corresponding functional classification to (A) and (B) and their respective comparisons.

Table S2. Differentially expressed carbon and amino acid/ nitrogen metabolism related proteins in *kinA*-overexpressed spores of *B. subtilis*

**Protein**	Log_2(M+/M-)	Log_2(M+/M-)	**function**	**product**
**Upregulation**				
		Both quantified in comparison with M- and WT+ spores		
**AroA**	2.45	1.89	biosynthesis of aromatic amino acids	3-deoxy-D-arabino-heptulosonate 7-phosphate synthase
**Quantified in comparison with M- spores**				
**AnsB**	1.14		aspartate degradation	L-aspartase
**Quantified in comparison with WT+ spores**				
**ThrC**		1.44	biosynthesis of threonine	threonine synthase
**Downregulation**				
**Both quantified in comparison with M- and WT+ spores**				
**AcsA**	-3.29	-3.45	utilization of acetate, fatty acids	acetyl-CoA synthetase
**ArtP**	-2.05	-1.17	arginine uptake	high affinity arginine ABC transporter (ATP-binding protein)
**BdhA**	-1.01	-1.46	overflow metabolism, fermentation	acetoine/ butanediol dehydrogenase
**CitZ**	-2.29	-2.05	TCA cycle	citrate synthase II
**DppA**	-3.11	-2.70	degradation of cell wall peptides	D-alanyl-aminopeptidase
**DppE**	-3.75	-1.58	uptake of dipeptides	dipeptide ABC transporter (dipeptide-binding protein)
**GlpK**	-2.33	-2.04	glycerol utilization	glycerol kinase
**MelA**	-1.19	-1.14	utilization of melibiose and raffinose family oligosaccharides (raffinose, stachyose)	alpha-galactosidase

**OppA**	-4.16	-2.53	initiation of sporulation, competence development	oligopeptide ABC transporter (binding protein)
**OppD**	-2.93	-1.42	initiation of sporulation, competence development	oligopeptide ABC transporter (ATP-binding protein)
**OppF**	-3.04	-1.84	initiation of sporulation, competence development	oligopeptide ABC transporter (ATP-binding protein)
**RbsD**	-2.61	-2.99	ribose uptake	ribose ABC transporter (membrane protein)
**RbsK**	-2.26	-2.28	ribose utilization	ribokinase
**Quantified in comparison with M- spores**				
**ArgC**	-1.51		biosynthesis of arginine	N-acetyl-g-glutamyl-phosphate reductase
**ArtM**	-1.52		arginine uptake	high affinity arginine ABC transporter (ATP-binding protein)
**ArtQ**	-1.66		arginine uptake	high affinity arginine ABC transporter (permease)
**BfmBAB**	-1.06		utilization of branched-chain keto acids	2-oxoisovalerate dehydrogenase (E1 beta subunit)
**BfmBB**	-1.12		utilization of branched-chain keto acids	2-oxoisovalerate dehydrogenase (E2 subunit, lipoamide acyltransferase)
**BkdAA**	-1.04		utilization of branched-chain keto acids	2-oxoisovalerate dehydrogenase (E1 alpha subunit)
**CitB**	-2.63		TCA cycle	aconitase, trigger enzyme
**GabD**	-1.26		utilization of gamma-amino butyric acid	succinate-semialdehyde dehydrogenase (NADP)
**GalE**	-1.37		galactose utilization	UDP glucose 4-epimerase
**GcvPB**	-1.94		glycine utilization	glycine decarboxylase (subunit 2)

**GcvT**	-1.69		glycine utilization	aminomethyltransferase (glycine cleavage system protein T)
**GlnH**	-2.49		glutamine uptake	glutamine ABC transporter (binding protein)
**GlpD**	-1.28		glycerol utilization	glycerol-3-phosphate dehydrogenase (menaquinone 7)
**HutG**	-4.32		histidine utilization	formiminoglutamate hydrolase
**HutI**	-4.89		histidine utilization	imidazolone-5-propionate hydrolase
**IolS**	-1.24		unknown, may be involved in myo-inositol catabolism	unknown
**KduI**	-1.07		utilization of galacturonic acid	5-keto-4-deoxyuronate isomerase
**LpdV**	-1.22		utilization of branched-chain keto acids	2-oxoisovalerate dehydrogenase (E3 subunit, dihydrolipoamide dehydrogenase)
**NagP**	-1.87		N-acetylglucosamine uptake and phosphorylation	N-acetylglucosamine-specific PTS, EIICB
**PutC**	-1.54		proline utilization	1-pyrroline-5-carboxylate dehydrogenase
**RbsB**	-1.90		ribose uptake	ribose ABC transporter (binding protein)
**YqiT**	-1.42		utilization of branched-chain keto acids	valine dehydrogenase, isoleucine dehydrogenase, L-leucine dehydrogenase
**Quantified in comparison with WT+ spores**				
**BglH**		-1.74	salicin utilization	phospho-beta-glucosidase
**FruK**		-1.26	fructose utilization	fructose-1-phosphate kinase
**HisB**		-1.13	biosynthesis of histidine	imidazoleglycerol-phosphate dehydratase
**IlvD**		-1.14	biosynthesis of branched-	dihydroxy-acid dehydratase

		chain amino acids	(2,3-dihydroxy-3-methylbutanoate, 2,3-dihydroxy-3-methylpentanoate)
**LicB**	-1.26	lichenan uptake and phosphorylation, control of LicR activity	lichenan-specific PTS, EIIB component
**OppC**	-1.52	initiation of sporulation, competence development	oligopeptide ABC transporter (permease)
**Pel**	-2.20	degradation of polygalacturonic acid	pectate lyase C
**ProA**	-1.00	biosynthesis of proline	glutamate-5-semialdehyde dehydrogenase
**RocA**	-2.74	arginine, ornithine and citrulline utilization	3-hydroxy-1-pyrroline-5-carboxylate dehydrogenase
**SdhA**	-2.55	TCA cycle	succinate dehydrogenase (flavoprotein subunit)
**SerC**	-1.25	biosynthesis of serine	3-phosphoserine aminotransferase
**YckE**	-1.19	utilization of aryl--glucosides	aryl--glucosidase
**YvdF**	-3.22	starch and maltodextrin utilization	glucan 1,4-alpha-maltohydrolase, neopullulanase, maltogenic amylase

Table S3. Differentially expressed proteins associated with coping of stress in *kinA*-overexpressed mutant spores of *B. subtilis*

Protein	Log_2(M+/M-)	Log_2(M+/WT+)	function	product
**Upregulation**				
**Both quantified in comparison with M- and WT+ spores**				
**yjqC**	1.66	2.49	protection of the spore	spore coat protein
Quantified in comparison with WT+ spores				
**skfA**		4.26	killing of sister cells	spore killing factor
**skfE**		1.13	export of the spore killing factor SkfA	ABC transporter (binding protein)
**ykzI**		3.44	unknown	unknown
**Downregulation**				
**Both quantified in comparison with M- and WT+ spores**				
**yfkD**	-1.39	-1.60	unknown	unknown
**yfhM**	-1.82	-1.46	survival of ethanol stress	epoxide hydrolase
**ydaF**	-1.83	-1.78	unknown	unknown
**Quantified in comparison with M- spores**				
**yvlB**	-1.15		unknown	unknown
**yuaG**	-2.22		involved in the control of membrane fluidity	membrane-associated scaffold protein
**yraA**	-1.31		detoxification of methylglyoxal	glyoxalase III-like enzyme
**yqfA**	-1.20		resistance against sublancin	flottilin-like protein
**yceD**	-1.44		required for survival of ethanol stress	unknown
**pbpE**	-2.33		endopeptidase	penicillin-binding protein PBP 4*
**katA**	-1.41		detoxification (degradation) of hydrogen peroxide	vegetative catalase

**gabD**	-1.26	utilization of gamma-amino butyric acid	succinate-semialdehyde dehydrogenase (NADP)
**Quantified in comparison with WT+ spores**			
**bceA**	-2.95	export of toxic peptides	bacitracin ABC transporter (ATP-binding protein)
**sdpI**	-1.99	protection against SdpC	immunity protein
**albC**	-3.83	export of antilisterial bacteriocin (subtilosin)	ABC transporter (ATP-binding protein)
**proA**	-1.00	biosynthesis of proline	glutamate-5-semialdehyde dehydrogenase
**rpmEB**	-1.07	survival of salt stress	accessory ribosomal protein
**yoaF**	-1.19	unknown	unknown
**ydhE**	-1.18	unknown	unknown
**srfAA**	-1.63	antibiotic synthesis, biocontrol of fungal growth	surfactin synthetase / competence
**phoR**	-1.93	regulation of phosphate metabolism	two-component sensor kinase
**phoP**	-1.55	regulation of phosphate metabolism (phoA, phoB, phoD, resABCDE, tag A-tagB, tagDEF, [tuaA-H])	two-component response regulator (OmpR family)
**ctsR**	-1.45	regulation of protein degradation	transcription repressor
**bceB**	-3.16	export of toxic peptides	bacitracin ABC transporter (permeas e)

Table S4. Differentially expressed proteins associated with coping of stress in *kinA* mutant cells of *B. subtilis* without induction

Protein	Log_2(M-/WT-)	function	product

AlbA	-4.65	antilisterial bacteriocin (subtilosin) production	radical S-adenosylmethionine enzyme
AlbC	-2.90	export of antilisterial bacteriocin (subtilosin)	ABC transporter (ATP-binding protein)
Ctc	-2.14	translation (under stress conditions)	ribosomal protein
Dps	-1.33	iron storage, survival of of stress conditions	mini-ferritin
GsiB	-3.74	response to water deficits	general stress protein
IolW	-1.40	utilization of scyllo-inosose	scyllo-inositol dehydrogenase
OhrB	-3.12	organic peroxide resistance	unknown
RsbV	-1.24	control of SigB activity	anti-anti-SigB
RsbW	-1.07	control of SigB activity	anti-sigma factor, protein serine kinase
SkfB	-1.35	maturation of spore killing factor	AdoMet radical sactisynthase
SkfC	-1.46	may be involved in spore killing	unknown
SkfE	-1.63	export of the spore killing factor SkfA	ABC transporter (binding protein)
YtxG	-1.14	unknown	unknown
YtxH	-1.13	unknown	unknown
YvyD	-1.71	dimerization of ribosomes in the stationary phase, protectioG a n against paraquat stress	ribosome hibernation promoting factor
YxxD	-1.05	inhibition of the cytotoxic activity of YxiD	antitoxin

Table S5. Differentially expressed proteins associated with amino/nitrogen metabolism in in *kinA* mutant spores of *B. subtilis* without induction

protein	Log_2(M-/WT-)	function	product
**AroB**	1.04	biosynthesis of aromatic amino acids	3-dehydroquinate synthase
**AroF**	1.55	biosynthesis of aromatic amino acids	chorismate synthase
**SerA**	1.35	biosynthesis of serine	phosphoglycerate dehydrogenase

**ArgC**	-1.02	biosynthesis of arginine	N-acetyl-g-glutamyl-phosphate reductase
**ARTP**	-1.32	arginine uptake	high affinity arginine ABC transporter (ATP-binding protein)
**BkdAB**	-1.01	utilization of branched-chain keto acids	2-oxoisovalerate dehydrogenase (E1 beta subunit)
**BkdB**	-1.04	utilization of branched-chain keto acids	2-oxoisovalerate dehydrogenase (E2 subunit, lipoamide acyltransferase)
**Buk**	-1.05	utilization of branched-chain keto acids	butyrate kinase
**oppA**	-1.45	initiation of sporulation, competence development	oligopeptide ABC transporter (binding protein)
**OPPD**	-1.57	initiation of sporulation, competence development	oligopeptide ABC transporter (ATP-binding protein)
**oppF**	-1.40	initiation of sporulation, competence development	oligopeptide ABC transporter (ATP-binding protein)
**NagP**	-1.21	N-acetylglucosamine uptake and phosphorylation	N-acetylglucosamine-specific PTS, EIICB
**PutC**	-2.14	proline utilization	1-pyrroline-5-carboxylate dehydrogenase
**PUTP**	-2.23	proline uptake	high affinity proline permease
