# Supplementary figures and images for: Artificial Sporulation Induction (ASI) by kinA Overexpression Affects the Proteomes and Properties of Bacillus subtilis Spores
Source: Int J Mol Sci. 2020 Jun 17;21(12):4315. doi: 10.3390/ijms21124315 (PMC7352945; doi:10.3390/ijms21124315)

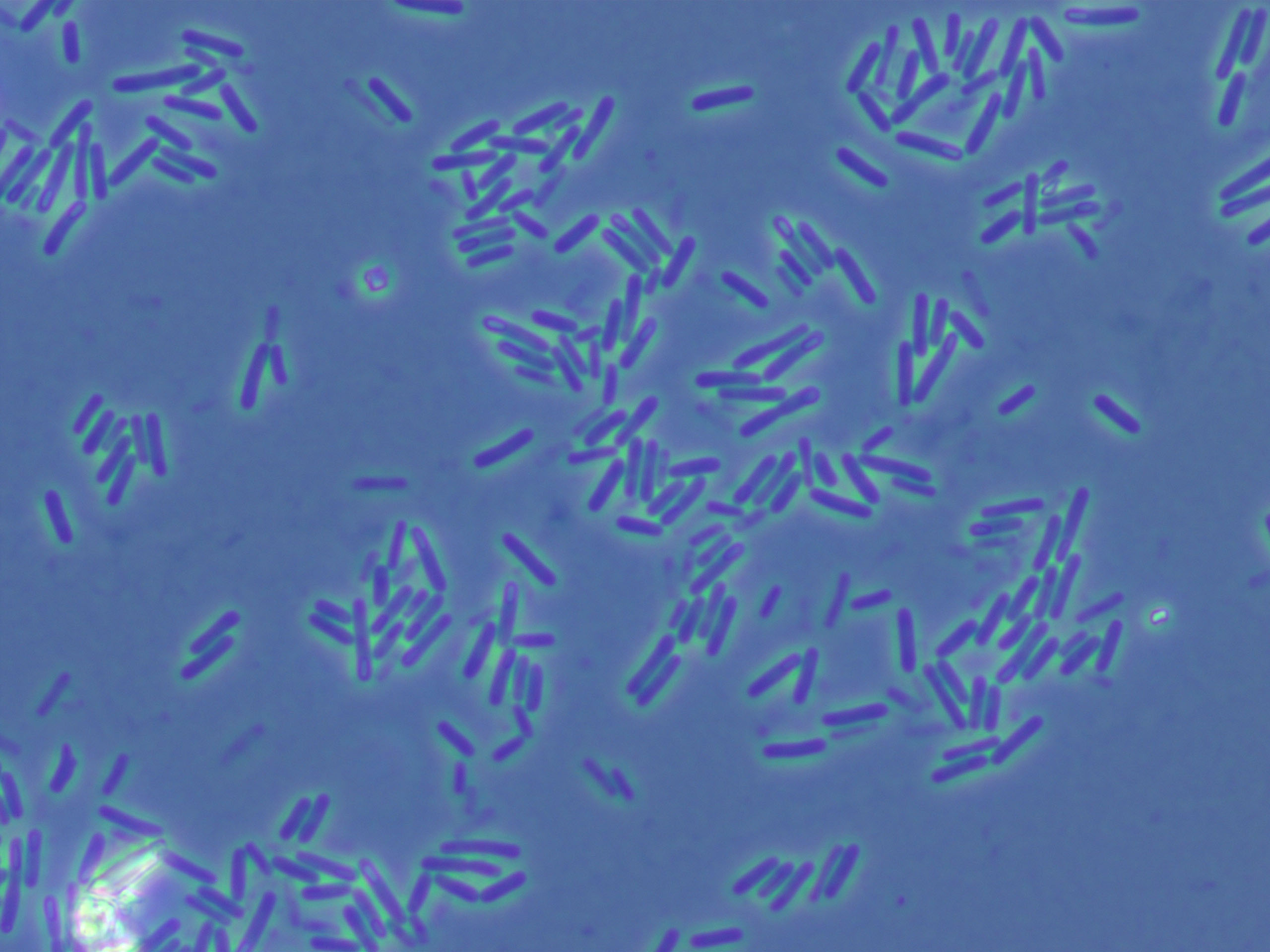

Supplement: Supplementary file 1 [file ijms-21-04315-s001.zip › File S1 Microscopic images/1887 for 3h IPTG.jpg]

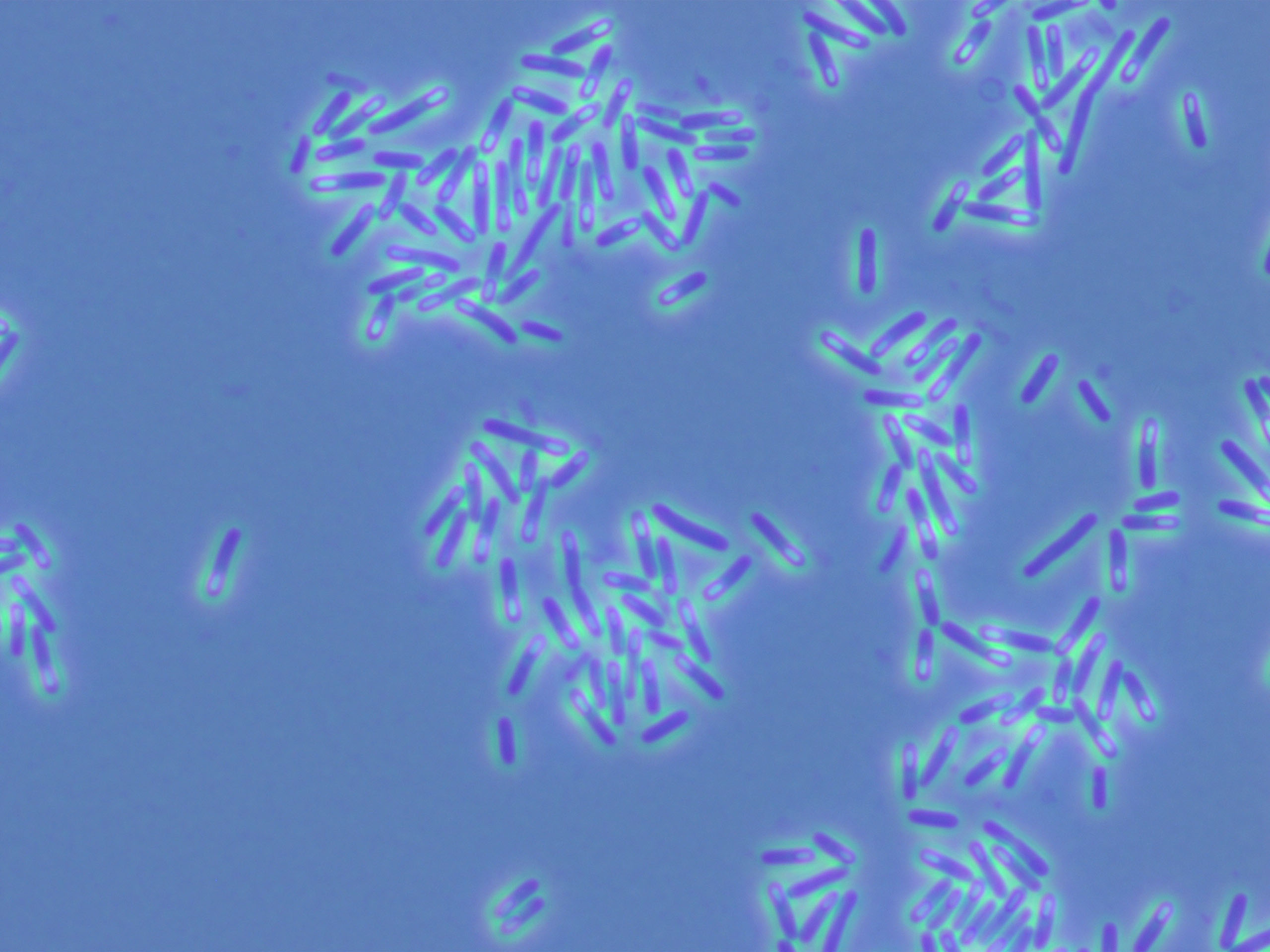

Supplement: Supplementary file 1 [file ijms-21-04315-s001.zip › File S1 Microscopic images/1887 for 4h IPTG.jpg]

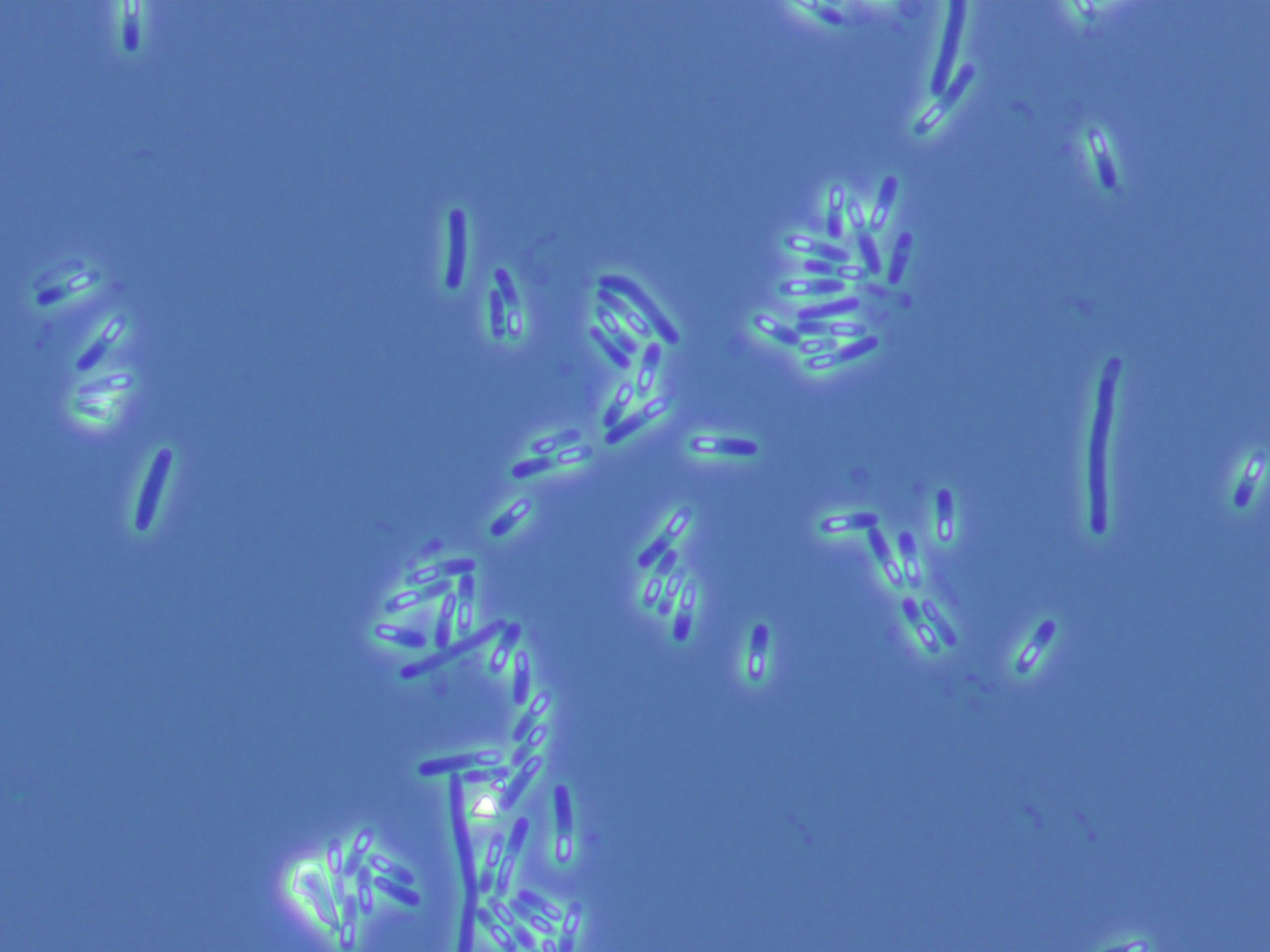

Supplement: Supplementary file 1 [file ijms-21-04315-s001.zip › File S1 Microscopic images/1887 for 5h IPTG.jpg]

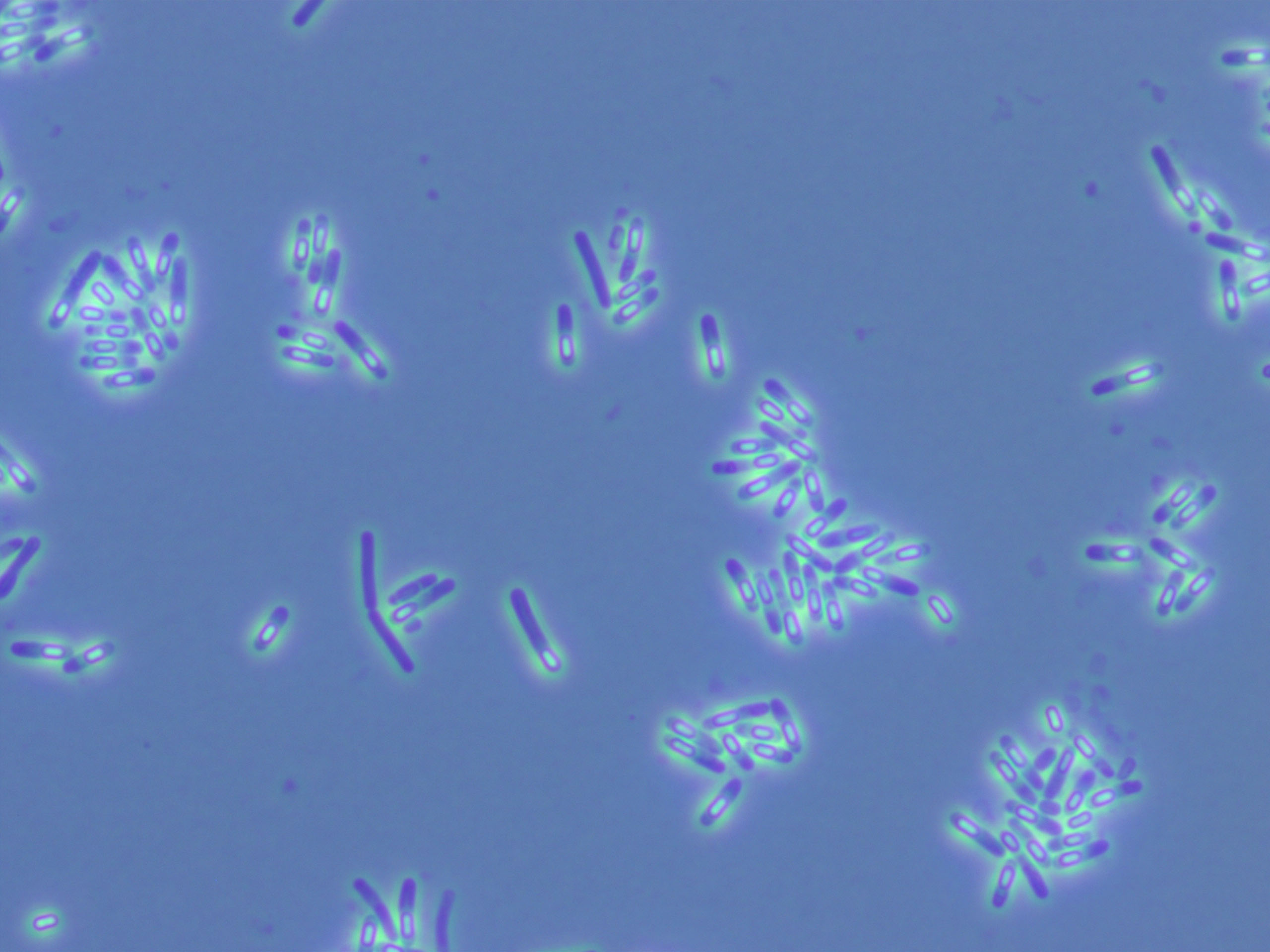

Supplement: Supplementary file 1 [file ijms-21-04315-s001.zip › File S1 Microscopic images/1887 for 6h IPTG.jpg]

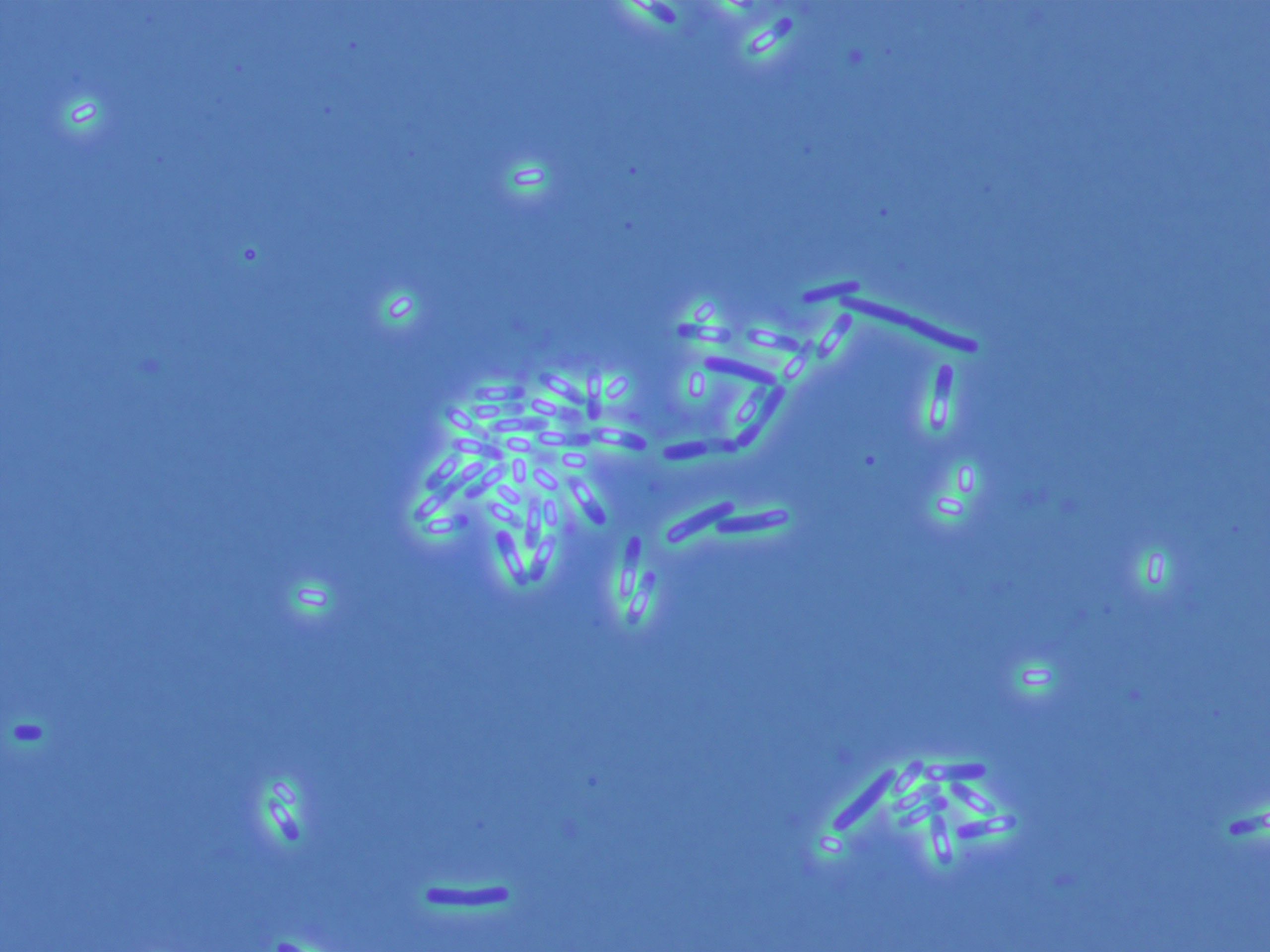

Supplement: Supplementary file 1 [file ijms-21-04315-s001.zip › File S1 Microscopic images/1887 for 7h IPTG.jpg]

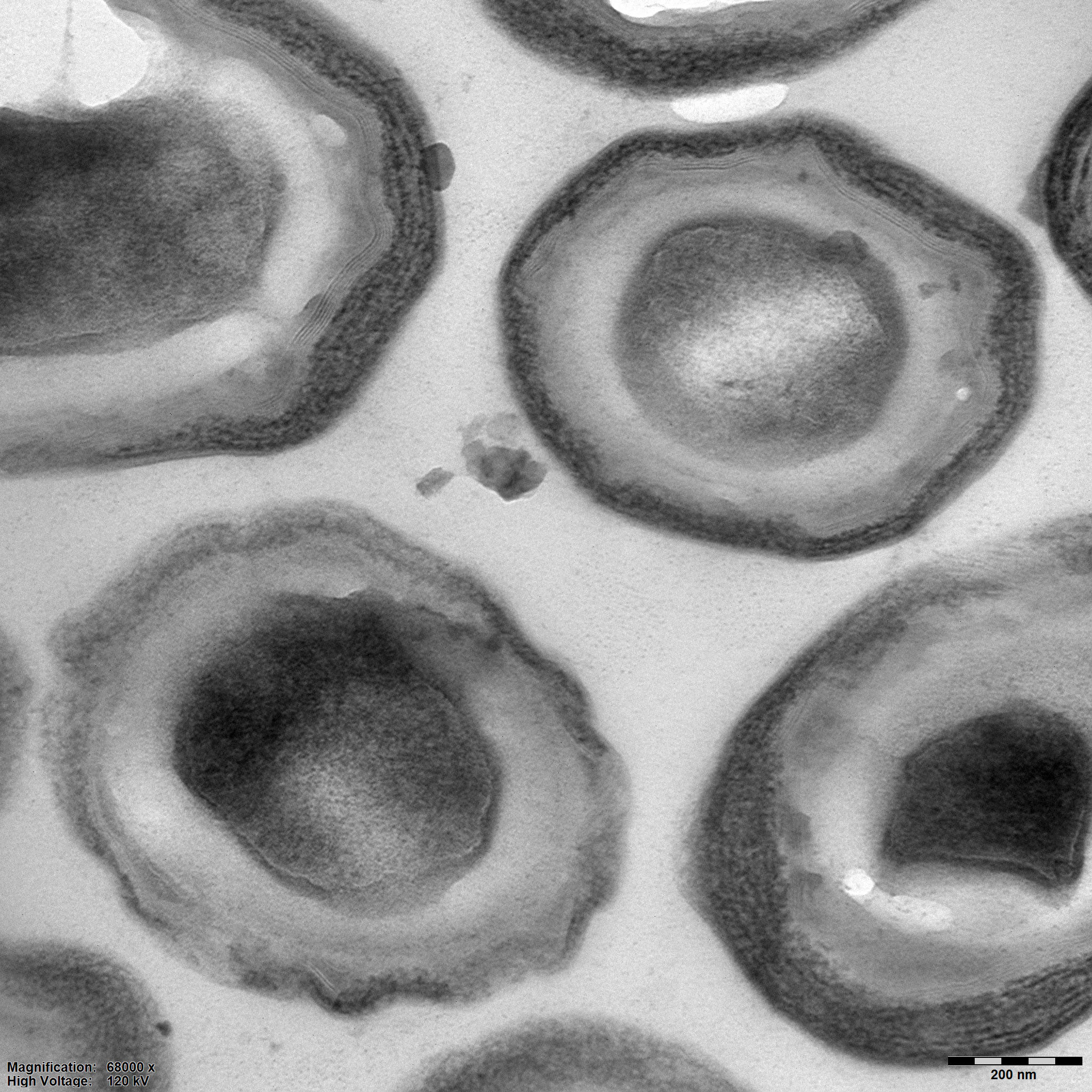

Supplement: Supplementary file 1 [file ijms-21-04315-s001.zip › File S2 TEM images/M+ spore.jpg]

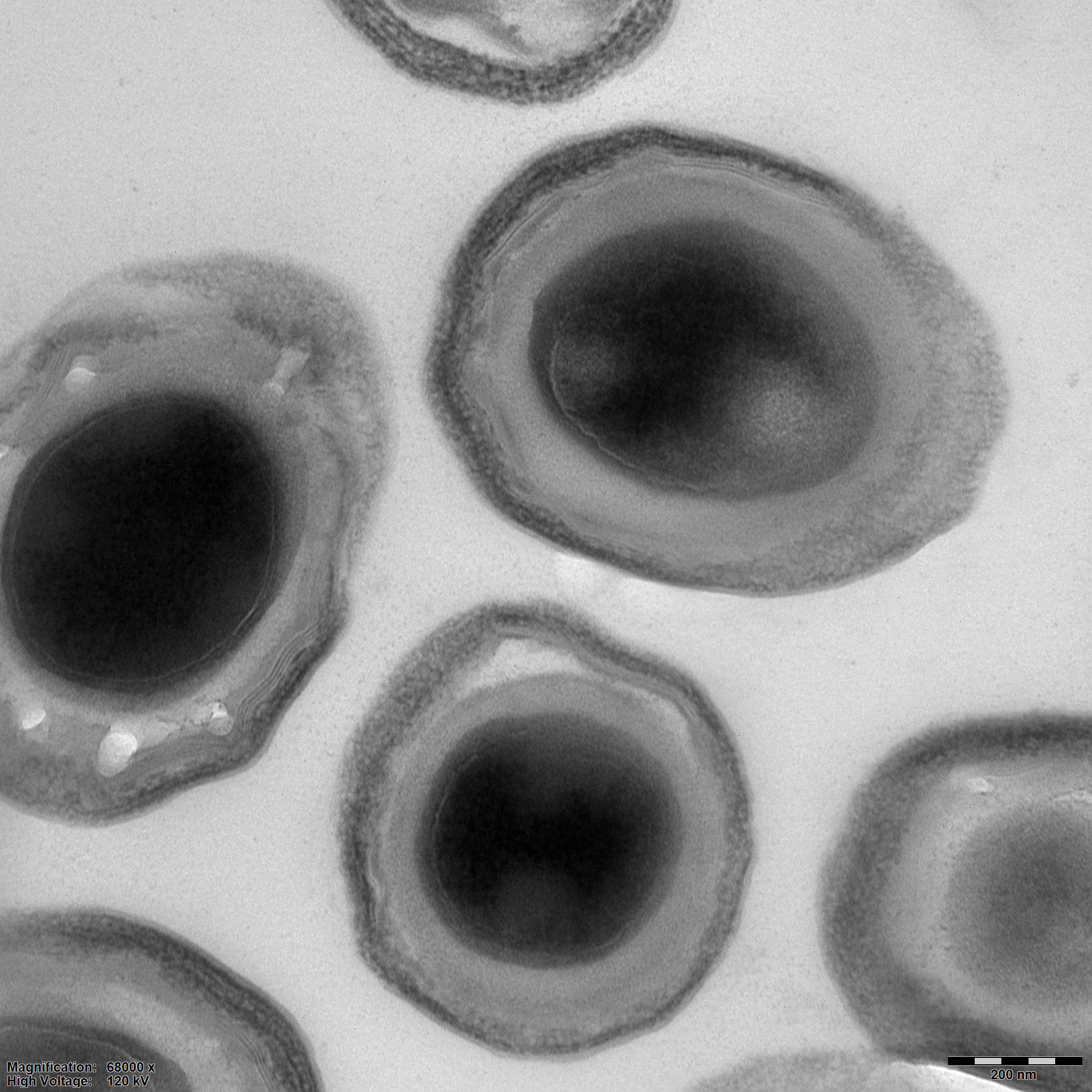

Supplement: Supplementary file 1 [file ijms-21-04315-s001.zip › File S2 TEM images/M- spore.jpg]

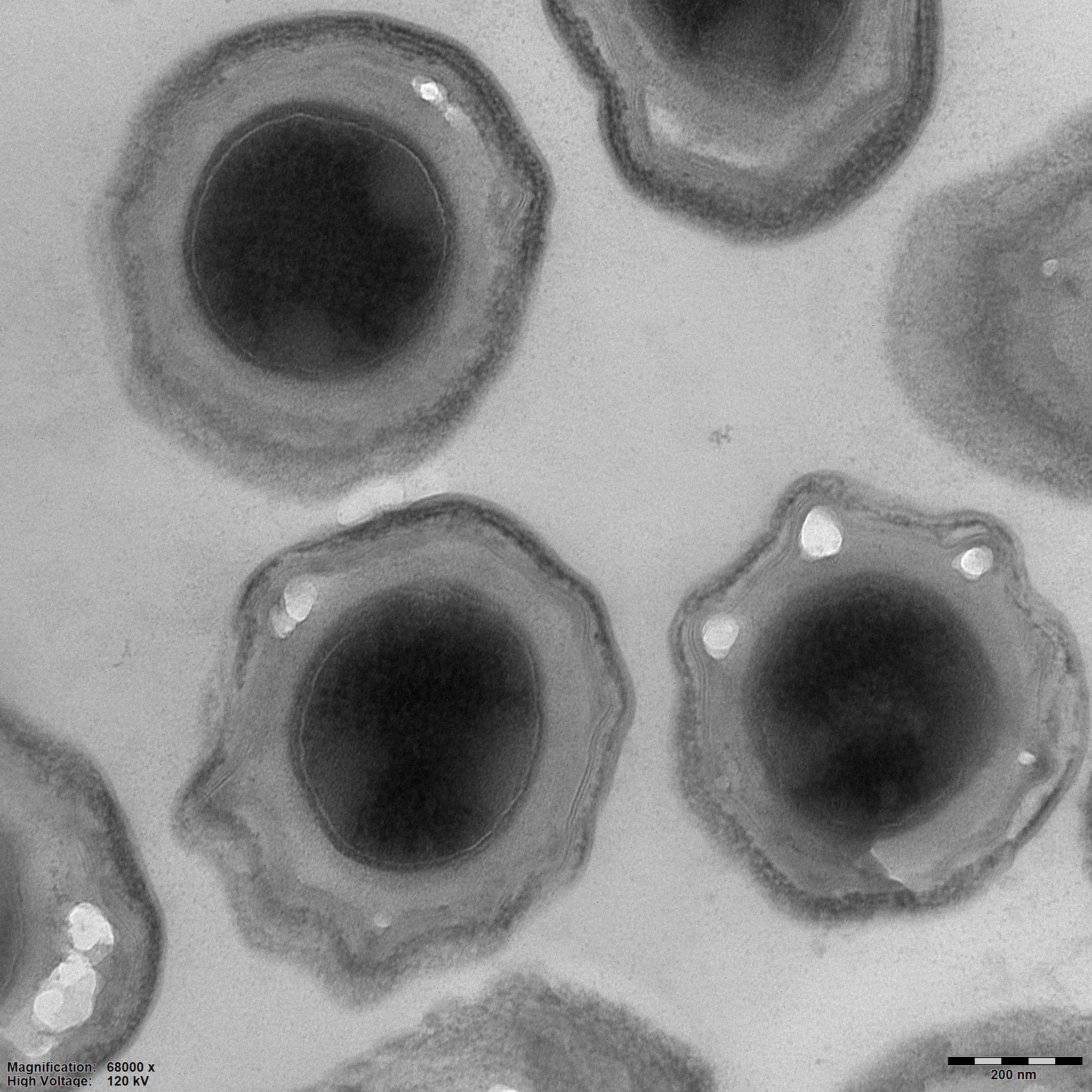

Supplement: Supplementary file 1 [file ijms-21-04315-s001.zip › File S2 TEM images/WT+ spore.jpg]

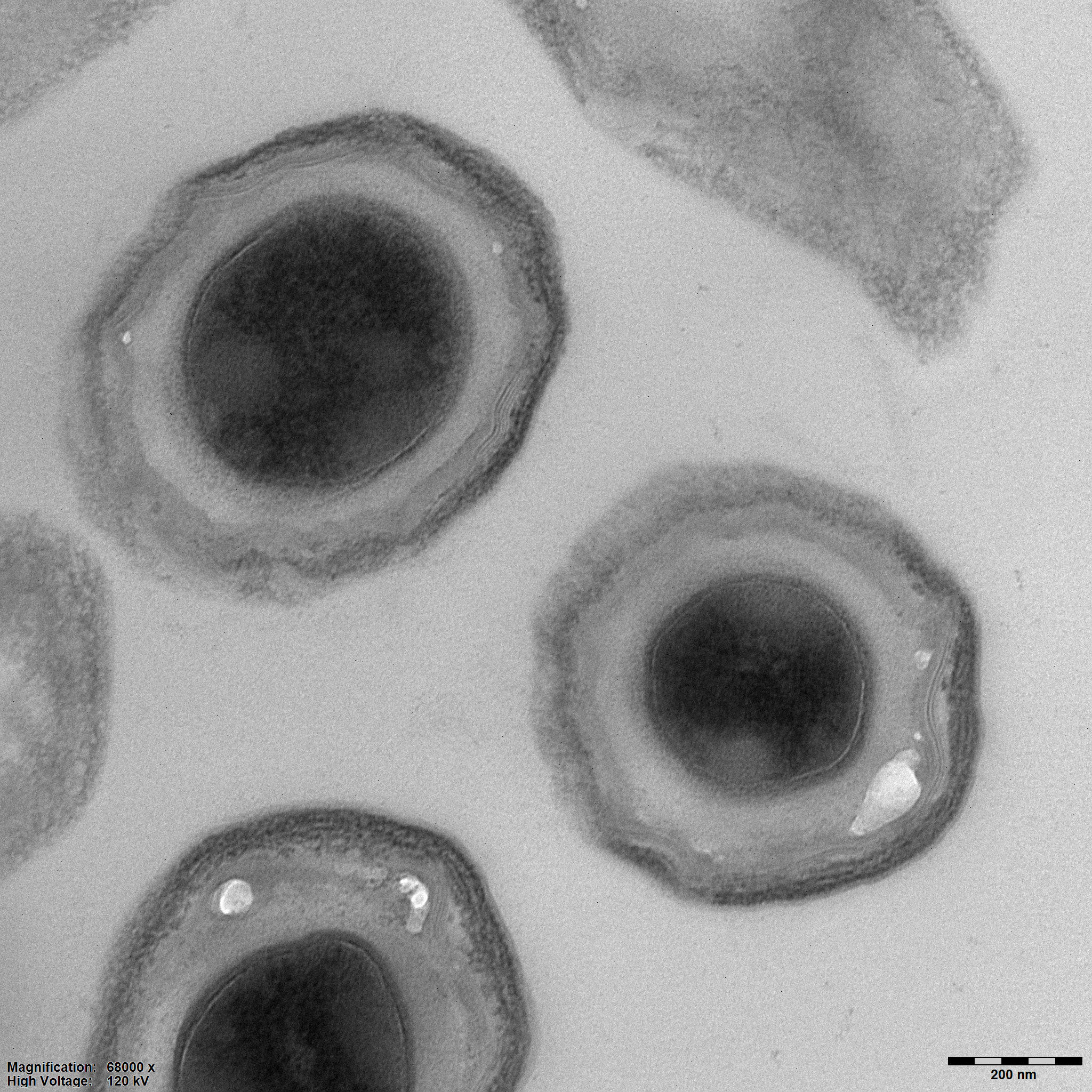

Supplement: Supplementary file 1 [file ijms-21-04315-s001.zip › File S2 TEM images/WT- spore.jpg]
